# Influence Mechanism of Chemically Modified Alumina on the Hydration of Gypsum-Based Self-Leveling Mortar

**DOI:** 10.3390/ma18214898

**Published:** 2025-10-26

**Authors:** Haozhe Ma, Meirong Zong, Nshuti Cedrick, Yuting Sun, Wenhao Wang, Xiancui Yan, Hui Liu, Pinghua Zhu, Minqi Hua

**Affiliations:** 1 Department of Civil Engineering, Changzhou University, Changzhou 213164, China; 13525413493@163.com (H.M.); kencedrick54@gmail.com (N.C.); s24210814016@smail.cczu.edu.cn (Y.S.); 19501141529@163.com (W.W.); yanxc@cczu.edu.cn (X.Y.); zph@cczu.edu.cn (P.Z.); 2 School of Civil Engineering & Architecture, Wuhan University of Technology, Wuhan 430070, China

**Keywords:** industrial by-product gypsum, self-leveling mortar, nano-active Al_2_O_3_, hydration mechanism

## Abstract

This study investigates the effect of γ-aminopropyltriethoxysilane (KH550)-functionalized nano-active Al_2_O_3_ (KH-Al) on the properties of gypsum-based self-leveling mortar (GSL) prepared from industrial by-product gypsum. First, the effects of incorporating KH-Al at dosages of 0.05%, 0.1%, 0.25%, 0.5%, and 1% on the fluidity, setting time, and mechanical properties of GSL were analyzed. Subsequently, using X-ray diffraction (XRD), hydration heat analysis, thermogravimetric analysis (TG), and scanning electron microscopy (SEM), the influences of the nanomaterial on the mortar’s morphology, hydration characteristics, and crystal forms of hydration products were thoroughly examined. Finally, by comparing the modified GSL with ordinary GSL, the mechanism of KH-Al’s action on GSL was elucidated. The results demonstrate that nano-active Al_2_O_3_ modified with KH550 exhibits excellent dispersibility in the GSL paste. As the dosage of KH-Al increases, both the fluidity and setting time of GSL decrease. Upon incorporating KH-Al, the mechanical properties of GSL initially improve and then decline, with optimal mechanical performance observed at a 0.5% KH-Al addition. However, when the KH-Al dosage exceeds 0.5%, excess nano-active Al_2_O_3_ causes nanoparticle agglomeration, which impedes the hydration process. The nucleation effect of KH-Al promotes the formation of CŜH_2_ and AFt, refines the crystals of hydration products, and enhances the phase transformation efficiency of the mortar. These findings indicate that KH-Al has significant potential to improve the mechanical strength and hydration kinetics of gypsum mortar and provide theoretical support for the application of nanomaterials in gypsum building materials.

## 1. Introduction

Gypsum-based building materials are widely used in the construction industry due to their advantages, including breathability, thermal insulation, resistance to hollowing and cracking, lightweight properties, and fire resistance [1]. These characteristics make them recognized as eco-friendly and sustainable building materials [2]. However, the rapid development of the construction industry has led to extensive exploitation of natural gypsum resources, resulting in a gradual depletion of reserves. Consequently, there is an urgent need to identify alternative resources to ensure the sustainable development of the construction sector [3]. The direct disposal and landfilling of industrial by-product gypsum—such as phosphogypsum (PG) and flue gas desulfurization gypsum (FGD)—not only consume significant land resources but also pose environmental risks [4]. From a sustainable development perspective, the resource utilization of industrial by-product gypsum is a top priority. Since the primary component of industrial by-product gypsum is calcium sulfate hemihydrate, which is chemically similar to natural gypsum, it represents a promising substitute for natural gypsum [5].

Gypsum-based self-leveling mortar (GSL) is an innovative functional building material designed for indoor flooring. It primarily utilizes calcium sulfate hemihydrate as the cementitious component and exhibits a certain degree of fluidity when mixed with water. Due to its advantages—such as low shrinkage, thermal insulation, and ease of application—GSL has garnered increasing attention in recent years [6]. Additionally, the incorporation of large quantities of industrial by-product gypsum in GSL is considered a sustainable approach to waste management. However, the presence of various impurities in industrial by-product gypsum often leads to performance issues in GSL, including inadequate mechanical properties and significant loss of fluidity [7]. To address these limitations, modifying GSL with low-energy, low-carbon-emission sulfoaluminate cement (SAC) offers an environmentally friendly solution. Previous studies have confirmed that SAC-modified β-hemihydrate gypsum primarily produces gypsum and ettringite (AFt) as hydration products, demonstrating excellent fluidity as well as high early-age and long-term compressive strength.

In recent years, emerging nanomaterials with unique microstructures and exceptional properties have been incorporated into cementitious systems to enhance mechanical performance, hydration mechanisms, and pore structure distribution [8]. These materials include carbon nanotubes [9], nano-graphene [10], nano-magnesium oxide [11,12], nano-silicon dioxide [13], among others. Similar to other nanomaterials, nano-Al_2_O_3_ exhibits strong nano-reinforcement capabilities; more importantly, the addition of nano-Al_2_O_3_ can accelerate gypsum hydration and promote the formation of hydration products.

Nano-Al_2_O_3_ is generally classified into two types: ordinary alumina (α-Al_2_O_3_) and activated alumina (γ-Al_2_O_3_). α-Al_2_O_3_ is formed by sintering aluminum precursors (γ-AlOOH) at high temperatures above 1200 °C. It is characterized by a dense lattice structure, low specific surface area, an inert surface, and minimal adsorption of hydroxyl groups. In contrast, γ-Al_2_O_3_ is produced by dehydrating γ-AlOOH at temperatures between 300 and 800 °C. It features a high specific surface area, partially vacant lattice sites, and a high concentration of surface hydroxyl groups (-OH) [14]. Roelofs et al. [15] discovered that γ-Al_2_O_3_ rapidly dissolves upon contact with aqueous solutions at pH values exceeding 9, resulting in a sharp increase in aluminum concentration until reaching a maximum at pH 11 (25 °C, 2.5 mmol/L). Furthermore, aluminum concentration is a critical factor governing AFT formation. The AFT content in the mortar system increases with higher proportions of alumina-containing reactants and total gypsum content, significantly affecting workability, compactness, and strength development [16,17,18,19]. Yu et al. [20] compared the effects of α-Al_2_O_3_ and γ-Al_2_O_3_ on the hydration process of gypsum mortar. Their study found that, in the gypsum paste system, γ-Al_2_O_3_ exhibited a higher dissolution rate and promoted the formation of hydration products such as ettringite and C-S-H gel. In contrast, α-Al_2_O_3_ had little to no effect on mortar hydration. Sun et al. [21] reported that nano-sized γ-Al_2_O_3_ not only enhanced the mechanical properties of gypsum slag cement but also improved the spatial distribution of ettringite, thereby preventing expansion caused by ettringite accumulation. In addition, nano-γ-Al_2_O_3_ rapidly releases Al^3+^ ions during initial dissolution, which consume Ca^2+^ and SO_4_^2−^ ions in the gypsum paste, leading to changes in the types of hydration products formed. This process results in the formation of different phases, such as ettringite (Ca_6_Al_2_(SO_4_)_3_(OH)_12_·26H_2_O, AFt), monosulfate aluminate hydrate (3CaO·Al_2_O_3_·CaSO_4_·12H_2_O, AFm), and monocarboaluminate (Ca_4_Al_2_(CO_3_)(OH)_12_·5H_2_O) [20,22]. An appropriate amount of these additional hydration products can further reduce the porosity of the mortar, optimize the pore structure, and minimize crack formation, thereby enhancing the mechanical properties and durability of the mortar [23].

However, due to its large specific surface area and the abundance of hydroxyl groups on its surface, γ-Al_2_O_3_ exhibits a strong tendency to self-aggregate in GSL [24]. When γ-Al_2_O_3_ is directly incorporated into the material, it is difficult to achieve uniform dispersion within the GSL matrix, preventing the full exploitation of the nanoparticles’ effects [25]. The γ-aminopropyltriethoxysilane (KH-550) reacts with the -OH groups on the γ-Al_2_O_3_ surface, transforming the hydrophilic inorganic surface into a hydrophobic organic surface. This surface functionalization not only reduces surface energy to suppress agglomeration but also introduces steric hindrance, thereby promoting the uniform dispersion of individual nanoparticles [26]. Therefore, in this study, we investigated the effects of varying dosages of KH-550-functionalized nano γ-Al_2_O_3_ on the macroscopic properties, hydration mechanisms, and microstructure of SAC-modified GSL. Through comprehensive analyses of fluidity, setting time, mechanical properties, X-ray diffraction (XRD), thermogravimetric and derivative thermogravimetric analysis (TG-DTG), scanning electron microscopy (SEM), and hydration heat, we elucidated the mechanism by which γ-Al_2_O_3_ influences GSL. To the best of our knowledge, this is the first study to publicly demonstrate the mechanism of γ-Al_2_O_3_ action on GSL, offering a novel approach to enhancing GSL performance. Furthermore, this research provides a new strategy for recycling industrial by-product gypsum and offers valuable insights for improving the performance of gypsum-based systems while addressing existing challenges.

## 2. Materials and Methods

### 2.1. Raw Materials

**Figure 1:**
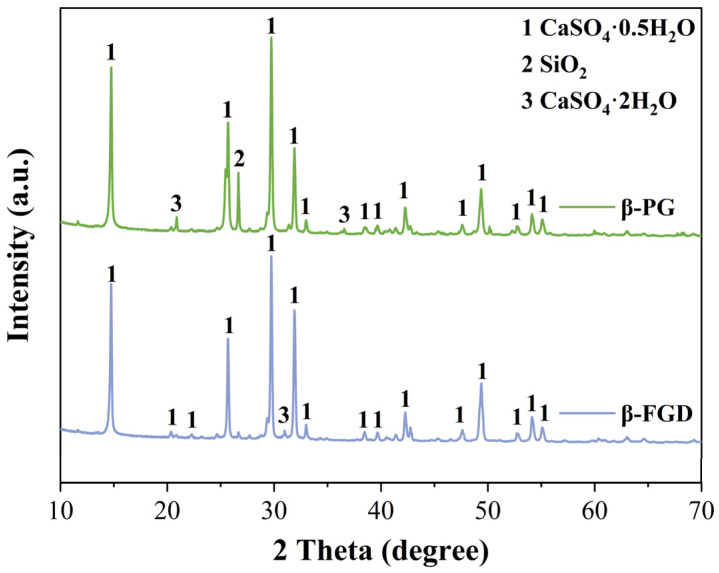
XRD patterns of β-PG and β-FGD.

In this study, β-PG and β-FGD, used for preparing GSL, were recycled from a phosphoric acid plant and Changzhou Hanchao Environmental Protection Technology Co., Ltd (Changzhou, China)., respectively. The XRD patterns of these two types of industrial by-product gypsum are shown in Figure 1, with their primary mineral phase identified as CaSO_4_·0.5H_2_O. The observed differences in the XRD patterns between β-PG and β-FGD are attributed to their distinct industrial origins and resulting impurity profiles. Specifically, the more pronounced CaSO_4_·2H_2_O peaks in β-PG indicate a higher residual dihydrate content, whereas the pattern for β-FGD reflects a higher purity hemihydrate with a significantly lower dihydrate presence. The characteristic SiO_2_ peak at approximately 26.6° is more intense in β-PG, consistent with its higher silicate impurity level. The mass fractions of the crystalline phases were estimated using the Rietveld refinement method. The analysis confirms the following typical composition ranges: β-PG consists of approximately 75–80% CaSO_4_·0.5H_2_O, 10–15% CaSO_4_·2H_2_O, and 5–10% SiO_2_; β-FGD comprises about 85–90% CaSO_4_·0.5H_2_O, 2–5% CaSO_4_·2H_2_O, and 3–8% SiO_2_. The chemical compositions of the different industrial by-product gypsums were analyzed using an X-ray fluorescence (XRF) spectrometer, as presented in Table 1. Sulfoaluminate cement (SAC) was used as a modifier for GSL, manufactured sand (40–70 mesh) served as the aggregate, and fly ash (Grade II) was employed as the mineral filler. All admixtures were powdered materials, including polycarboxylate superplasticizer (PCE, Model: ZJ-PC8310, Product Code: 24010674) and protein retarder (PR, Model: ZJ-G22, Product Code: 24010754), which are used to ensure the fluidity required for construction while reducing water consumption and extending setting time, thereby improving strength and enhancing construction performance. Hydroxypropyl methylcellulose (HPMC) water retention agent (Model: ZJ-400E, Product Code: 24022873) is employed to retain water in GSL, preventing mortar segregation. Additionally, defoamer (DA, Model: ZJ-D130, Product Code: 24030987) is used to eliminate bubbles introduced during mixing, thus improving the compactness and surface flatness of the mortar. All admixtures were of analytical grade and required no further purification.

### 2.2. Preparation of KH550-Functionalized Nano-γ-Al_2_O_3_ Modified Mortar

**Figure 2:**
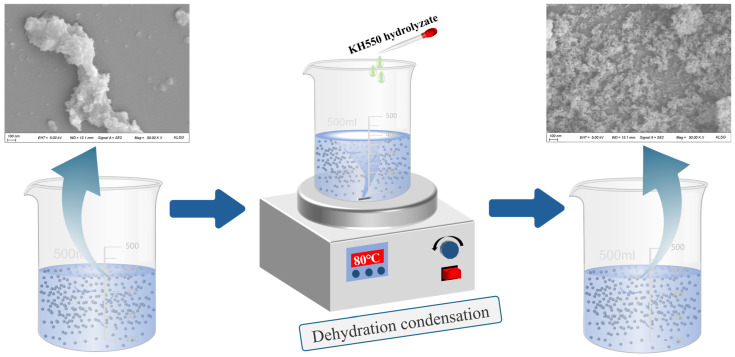
Preparation process and surface functionalization mechanism of KH-Al nanoparticles via KH550.

**Figure 3:**
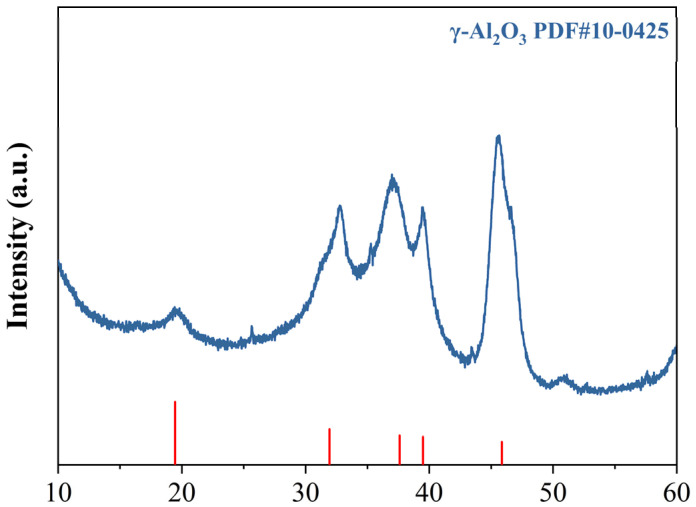
XRD patterns of KH-Al.

The preparation method for KH550-functionalized nano-γ-Al_2_O_3_; was optimized through preliminary experiments, and the detailed procedure is illustrated in Figure 2. First, weigh KH550, deionized water, and absolute ethanol separately, then mix them at a mass ratio of 1:1:10. Stir the mixture with a magnetic stirrer for 60 min to ensure complete hydrolysis of KH550. Next, disperse nano-γ-Al_2_O_3_ in absolute ethanol; once the nano-mineral powder is fully wetted, ultrasonicate the suspension for 30 min to achieve a concentration of 2 wt%. Add 200 mL of this nano-mineral suspension and 85 mL of deionized water to a magnetic stirrer. At 80 °C, slowly add 15 mL of the hydrolyzed KH550 solution dropwise while stirring continuously for 60 min. During this process, the silanol groups in the KH550 molecules replace some hydroxyl groups on the surface of the nano-mineral powder, providing steric hindrance to the particles. After the reaction, centrifuge the mixture at 8000 rpm for 5 min to separate the nanoparticles. Then, wash the precipitate with absolute ethanol four times to remove unreacted silane coupling agent and other impurities, yielding KH550-functionalized nano-γ-Al_2_O_3_, hereafter referred to as KH-Al. The diffraction peaks of the modified KH-Al show good agreement with the standard γ-Al_2_O_3_ PDF card, and no significant impurity peaks are observed, as shown in Figure 3.

The mixed proportions of different GSL samples are presented in Table 2, with a consistent water-to-binder ratio of 0.4 for all samples. First, mix KH-Al with PCE, then add the mixture to water and use an ultrasonic disperser to sonicate for 30 min to ensure proper dispersion of the nanomaterial. Weigh 2 kg of dry materials (including gypsum powder, fly ash, and manufactured sand) and mix them at low speed in a planetary mixer for 30 s. Next, add the nano-mineral powder dispersion and admixtures to the planetary mixer and continue high-speed stirring for 60 s. Immediately after mixing, remove the fresh mortar from the mixer and measure its fluidity and setting time. According to the Chinese standard JC/T 1023-2021 [27], divide the fresh mortar into two groups and pour them into mold boxes (40 mm × 40 mm × 160 mm) pre-coated with a mold release agent. Cure the specimens under standard conditions (temperature: 20 ± 5 °C, relative humidity: 65 ± 10%). Demold the specimens after curing for (24.0 ± 0.5) hours and perform compressive and flexural strength tests on one group after 24 h. Cure the other group under standard conditions for 28 days, then place it in an oven at (40 ± 0.5) °C until a constant weight is achieved (approximately 2 days). After the specimens cool to room temperature, conduct the strength tests.

### 2.3. Test Methods

#### 2.3.1. Fluidity

Immediately pour the fresh mortar into a fluidity test mold (inner diameter 40 mm, height 50 mm). Then, place the mold in the center of the test plate, lift it vertically to a height of 50–100 mm above the test plate within 2 s, and hold this position for 10–15 s. After allowing the mortar to flow freely for 4 min, measure the arithmetic mean of its diameters in two perpendicular directions; this value represents the initial fluidity. After letting the newly mixed mortar stand for 30 min, stir it at high speed for 30 s using a planetary mixer before conducting the fluidity test. The arithmetic mean of its diameters in two perpendicular directions at this stage is the 30-min fluidity.

#### 2.3.2. Setting Time

The setting time of the mortar was tested in accordance with the requirements of GB/T 17669.4 [28]. A Vicat apparatus (Shanghai Luda, Shanghai, China) was used to measure the initial and final setting times of the GSL. Tests were conducted every 10 min during the first hour and every 5 min thereafter. The initial setting time was reached when the freely falling Vicat needle failed to penetrate the mortar to the glass base plate. The final setting time was reached when the needle’s penetration depth into the mortar was no more than 1 mm.

#### 2.3.3. Mechanical Properties

The compressive and flexural strengths were tested in accordance with GB/T 17669.3 [29]. For each batch of GSL samples, six mortar specimens measuring 40 mm × 40 mm × 160 mm were prepared and demolded after curing for 1 day and 28 days. A cement mortar compressive and flexural testing machine was used to measure the flexural strengths of the specimens at both 1 day and 28 days. After the flexural test, the specimens were cut into cubic samples measuring 40 mm × 40 mm × 40 mm, and the compressive strength test was subsequently performed.

#### 2.3.4. XRD Analysis

Different GSL samples were ground into powders with particle sizes less than 100 μm and dried in an oven at 45 °C for 6 h. The phase composition of the various GSL samples was analyzed using X-ray diffraction (XRD; Rigaku Corporation, Tokyo, Japan) with a scanning speed of 5°/min and a scanning angle (2θ) range of 5° to 80°.

#### 2.3.5. TG-DTG

The weight loss of the samples (10 mg each) was measured using a thermogravimetric analyzer (TG 209 F3, Netzsch, Selb, Germany). The heating rate was set to 10 °C/min, with a temperature range from 50 °C to 300 °C, under a nitrogen (N_2_) atmosphere. Thermogravimetric (TG) and derivative thermogravimetric (DTG) analyses accurately identify weight loss at each stage through mass loss steps and derivative peaks (DTG curves), providing valuable information for characterizing the quantity of hydration products.

#### 2.3.6. Hydration Heat Evolution

At 20 °C, an isothermal conduction calorimeter (TAM Air, Houston, TX, USA) was used to measure the heat release during the hydration of mortar over 24 h. The results were employed to characterize the degree of hydration of different GSL samples. Each component material was weighed proportionally; after reaching thermal equilibrium, deionized water was added, followed by homogenization for 2 min. The test data were then automatically recorded under constant temperature conditions.

#### 2.3.7. SEM/EDS Analysis

The microstructure of GSL was examined using a field emission scanning electron microscope (FE-SEM, SUPRA55, Zeiss, Oberkochen, Germany) at an accelerating voltage of 5–15 kV.

#### 2.3.8. FT-IR Analysis

Different GSL samples were ground into powders. Then, 20 mg of each GSL sample was mixed with 1 g of potassium bromide (KBr) powder, followed by thorough grinding and tableting. A Fourier transform infrared spectrometer (FT-IR, IS50, Thermo Fisher Scientific, Waltham, MA, USA) with a scanning range of 4000–400 cm^−1^ was used to analyze the distribution of chemical functional groups in the samples.

## 3. Results and Discussion

### 3.1. Effect of KH-Al on Fluidity and Setting Time of GSL

**Figure 4:**
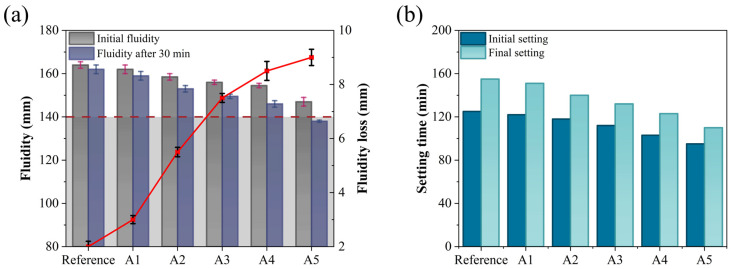
Effects of KH-Al dosage on (**a**) fluidity and (**b**) setting time of GSL.

Fluidity and setting time are critical parameters in the construction process of GSL, as they directly influence construction efficiency, ground formation quality, and subsequent service performance. Figure 4 examines the effects of varying KH-Al dosages on the fluidity and setting time of GSL.

According to the requirements of Chinese Standard JC/T 1023-2021 [27], the initial fluidity and 30-min fluidity of GSL should not be less than 140 mm. As shown in Figure 4a, with increasing KH-Al dosage, both the initial fluidity and 30-min fluidity of GSL exhibit a decreasing trend, while the 30-min fluidity loss increases. Compared to the control group mortar, the initial fluidity of Sample A5 decreases by 17 mm, the 30-min fluidity decreases by 24 mm, and the fluidity loss increases from 2 mm to 9 mm. The reduction in fluidity is attributed to KH-Al’s large specific surface area, which preferentially absorbs free water in the mortar, thereby reducing the amount of free water available for particle dispersion and friction reduction within the mortar system. This results in a significant increase in mortar consistency, ultimately manifesting as decreased fluidity [30]. Furthermore, when the KH-Al dosage is further increased, the nanoparticles transition from a nanoscale dispersed state to larger aggregates within the mortar. These aggregates create physical barriers between gypsum particles, impeding their relative sliding, leading to poor void filling and a substantial increase in flow resistance, which further reduces mortar fluidity. However, at a KH-Al dosage of 0.5%, the fluidity of GSL still meets the performance requirements specified in Chinese Standard JC/T 1023-2021. This is because the silanol groups of KH550 molecules chemically react with the hydroxyl groups (-OH) on the surface of nano-alumina, forming strong covalent bonds that convert the hydrophilic inorganic surface into a hydrophobic organic surface. This modification reduces the surface energy of the particles and fundamentally inhibits agglomeration. The modified particles repel each other due to their hydrophobic surfaces, promoting more uniform dispersion within the mortar. Consequently, the nanoparticles exert a dispersion and lubrication effect to some extent, mitigating their adverse impact on fluidity.

The setting time of GSL (typically requiring an initial setting time of ≥60 min and a final setting time of ≤360 min) directly influences the construction cycle and strength development. An appropriate setting time ensures sufficient working time and promotes early strength gain. As shown in Figure 4b, increasing the dosage of KH-Al reduces the initial setting time of GSL from 125 min to 95 min and the final setting time from 155 min to 110 min. Consequently, the interval between the initial and final setting times decreases from 30 min to 15 min. This reduction in setting time is attributed to KH-Al, which provides nucleation sites for the hydration of gypsum dihydrate (CaSO_4_·2H_2_O, CŜH_2_), thereby accelerating the transition of GSL from a plastic to a hardened state [31]. Additionally, Al^3+^ ions released from KH-Al may react with Ca^2+^ and SO_4_^2−^ ions, further accelerating the overall hardening process [22].

### 3.2. Effect of KH-Al on Mechanical Properties of GSL

**Figure 5:**
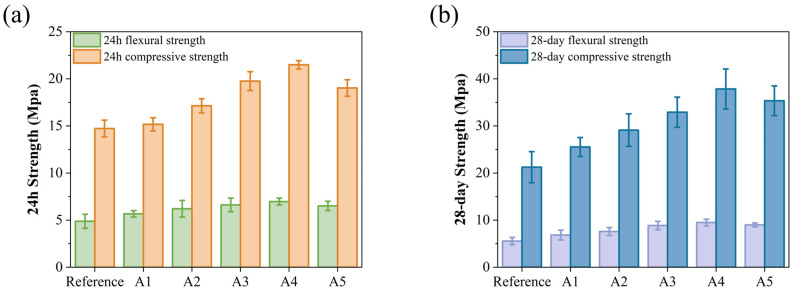
Effects of KH-Al dosage on mechanical properties of GSL: (**a**) 24 h, (**b**) 28 days.

The incorporation of KH-Al results in an increase in early strength, as shown in Figure 5a. The compressive and flexural strengths of group A4 reach 6.97 MPa and 21.49 MPa, respectively, representing increases of 42.83% and 45.89% compared to the control group (4.88 MPa and 14.73 MPa). KH-Al significantly enhances the early strength of GSL, an effect that also contributes to improved 28-day strength. As illustrated in Figure 5b, among all samples, group A4 exhibits the best 28-day mechanical properties, with flexural and compressive strengths increased by 70.86% and 78.12%, respectively. This improvement is attributed to KH-Al’s high surface energy, which substantially reduces the nucleation energy barrier of crystals and refines crystal particle size. Furthermore, KH550 modification ensures that the nanoparticles are uniformly dispersed within the gypsum matrix as single particles or small aggregates, thereby maximizing the size effect of the nanomaterials [32]. Upon dispersion, KH-Al penetrates the micropores and interfacial gaps between CŜH_2_ crystals, directly reducing pore volume and promoting strength development. This also confirms that nano-minerals modified with KH550 achieve a more uniform distribution within the mortar matrix. The decline in mechanical properties observed in group A5 is due to the excessive addition of KH-Al. KH-Al tends to absorb moisture and agglomerate within the paste, causing the nanoparticles to lose their filling capacity and reducing the number of nucleation sites [33]. Previous studies have reported that increasing the nano-γ-Al_2_O_3_ content leads to significant secondary agglomeration in the paste, which adversely affects the microstructure [31]. These results confirm that the efficacy of KH550 surface modification is dosage-dependent. When the nanoparticle concentration exceeds the dispersant’s capacity to maintain steric stabilization, the protective steric barrier is compromised, leading to re-agglomeration. This causes the system to revert to a state resembling the poor performance of unmodified agglomerates [34,35]. Thus, the fundamental mechanistic function of KH550 is to substantially raise the dosage threshold at which re-agglomeration occurs. This shift allows a greater proportion of nanoparticles to remain individually dispersed, maximizing their contribution to microstructural densification and strength development.

### 3.3. XRD Analysis

**Figure 6:**
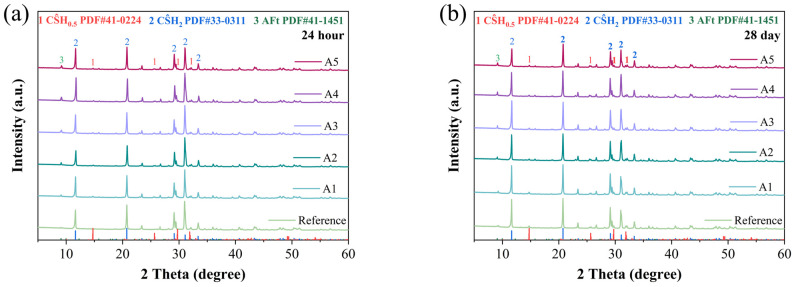
XRD patterns under different KH-Al dosages: (**a**) 24 h, (**b**) 28 days.

The crystalline phases of hydration products in different GSL samples were analyzed by X-ray diffraction (XRD), and the results are shown in Figure 6. It can be observed that the primary hydration products in the various GSL samples are CŜH_2_ and AFt. From Figure 6a,b, it is evident that with increasing KH-Al dosage, the intensity of the ettringite (AFt) peak gradually increases after 24 h and 28 days of curing. In the early stage of hydration, CŜH_0.5_ transforms into CŜH_2_ according to Equation (1). AFt is the hydration product of C_4_A_3_Ŝ (Equation (2)). As the hydration reaction progresses, Al_2_O_3_ in the GSL system is activated and dissolved, generating aluminate ions (Equation (3)). The SO_4_^2−^ and Ca^2+^ ions in GSL react with these aluminate ions via Equation (4), leading to a further increase in AFt content. Therefore, the incorporation of KH-Al promotes the formation rate of AFt, and no formation of monosulfate aluminate (AFM) is observed. For samples containing KH-Al, the intensity of the diffraction peak of CŜH_2_ exhibits a trend of initially increasing and then decreasing on the 1st and 28th days. This occurs because when KH-Al is incorporated into the mortar at a low, well-dispersed dosage, its fine particles can uniformly distribute throughout the gypsum paste, thereby promoting the hydration process of the gypsum. However, excessive nanoparticle aggregation or poorly dispersed particles occupy hydration space, hindering the dissolution of CŜH_0.5_ and the growth of CŜH_2_ crystals [36]. Additionally, the further formation of AFt consumes Ca^2+^ and SO_4_^2−^ ions in the GSL system, which reduces the formation of CŜH_2_ crystals.

(1) $${C\hat{S}H}_{0.5}+1.5H\to {C\hat{S}H}_{2}$$

(2) $$C_{4}A_{3}\hat{S}+2C\hat{S}H_{2}+34H\to C_{6}A{\hat{S}}_{3}H_{32}+2AH(\mathrm{gel})$$

(3) $$A+3H+2OH^{-}\to 2Al{\left(OH\right)}_{4}^{-}$$

(4) $$6Ca^{+}+2Al{\left(OH\right)}_{4}^{-}+3SO_{4}^{2-}{+4\mathrm{OH}}^{-}+26H\to C_{6}A{\hat{S}}_{3}H_{32}$$

### 3.4. TG-DTG Analysis

**Figure 7:**
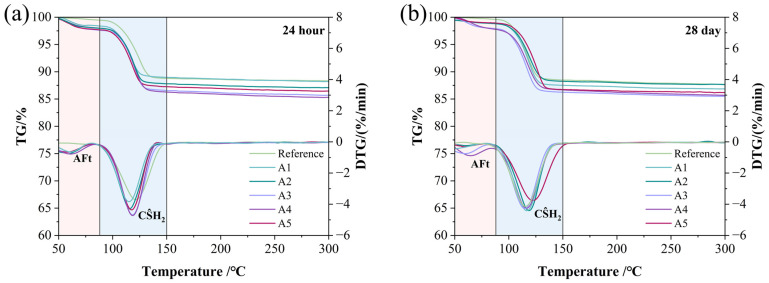
TG-DTG patterns under different KH-Al dosages: (**a**) 24 h, (**b**) 28 days.

TG-DTG analysis was used to determine the specific contents of the main hydration products in different GSL samples. All GSL samples exhibited two mass loss peaks, corresponding to the thermal decomposition of AFt (at 75 °C) and CŜH_2_ (at 125 °C), respectively. The thermal decomposition reactions are presented in Equations (5) and (6). With the incorporation of KH-Al, the mass loss of GSL increased to varying degrees at both 24 h (Figure 7a) and 28 days (Figure 7b). This indicates that doping with KH-Al significantly increases the proportions of AFt and CŜH_2_. The yield of hydration products was calculated based on the amount of crystalline water lost by GSL at different temperature stages, as shown in Equation (7). As shown in Table 3, the yield of hydration products at each stage increases with the addition of KH-Al. Specifically, Sample A4 exhibits the highest hydration product yield (total yield of AFt and CŜH_2_), with increases of 10.80% at 24 h and 7.21% at 28 days, respectively. This is attributed to the nanoparticles providing abundant nucleation sites for AFt and CŜH_2_, thereby accelerating the hydration reaction and enhancing its completeness. This also indicates that the strength enhancement resulting from the incorporation of KH-Al aligns with the general principle, but it follows different chemical pathways compared to other nanomaterials. While the strength gain from nano-SiO_2_ is primarily attributed to the pozzolanic formation of additional C-S-H gel [37], and nano-MgO contributes through micro-filling and the formation of an expansive brucite network [38], the main role of KH-Al in the GSL system is to promote the formation of a dense network of AFt and interlocked CŜH_2_ crystals. This suggests that the selection of nanomaterials should be system-specific; for gypsum-based systems rich in sulfate, nano-γ-Al_2_O_3_ provides a more chemically compatible and efficient pathway to microstructural densification.

(5) $$C\hat{S}H_{2}\to C\hat{S}+2H$$

(6) $$C_{3}A\cdot 3C\hat{S}\cdot 32H\to C_{3}A\cdot C\hat{S}\cdot 12H+2C\hat{S}+20H$$

(7) $$W_{i}=\Delta W\times \frac{M_{i}}{nM_{water}}\times 100\%$$

### 3.5. Heat Flow Analysis of GSL Hydration Process

**Figure 8:**
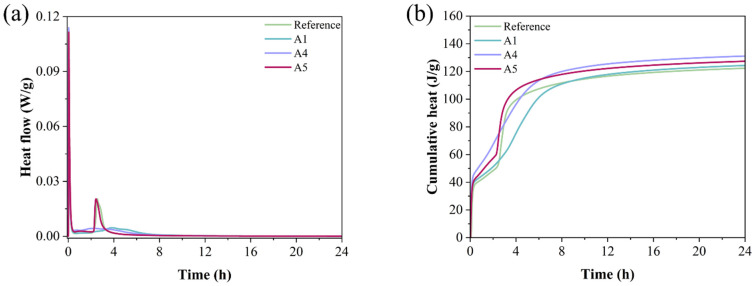
Heat release curves of mortars with different KH-Al dosages in the first 24 h: (**a**) Heat flow rate, (**b**) Cumulative heat release.

Figure 8 illustrates the heat release during the hydration of various GSL samples. As shown in Figure 8a, during the early stage of hydration, the heat flow curve of the GSL sample reaches its peak when the KH-Al dosage is 0.5%. This is because KH-Al, uniformly dispersed within the gypsum paste, provides numerous evenly distributed nucleation sites. This characteristic accelerates the hydration reaction rate of GSL, resulting in greater heat generation per unit time, which is reflected macroscopically as an increased peak in the heat flow curve. The second exothermic peak corresponds to the formation of a substantial amount of hydration products. An optimal dosage of KH-Al ensures a more uniform release of hydration heat throughout the reaction process, leading to a reduction in the peak value of the second exothermic peak. However, excessive KH-Al causes the hydration reaction of GSL to become overly concentrated and significantly shortens the setting time, which is detrimental to strength development and on-site construction [39]. Figure 8b shows that Sample A4 exhibits the highest cumulative heat release, indicating a relatively high degree of hydration. Therefore, the addition of KH-Al effectively promotes the hydration of the GSL system.

### 3.6. SEM Analysis

**Figure 9:**
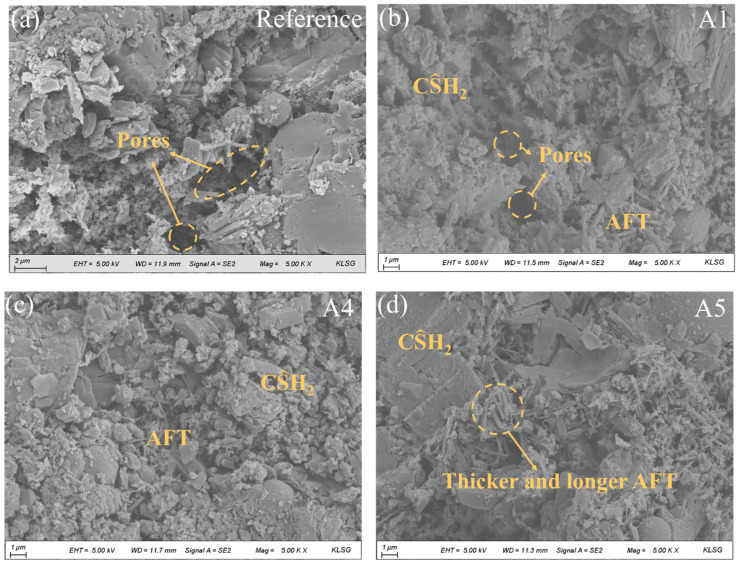
SEM images of mortars with different KH-Al dosages after 28 days of curing, (**a**) 0%; (**b**) 0.125%; (**c**) 0.5%; (**d**) 1%.

SEM images of various GSL samples after 28 days of hydration are presented in Figure 9. Without the addition of any nano-mineral powder, the degree of hydration in GSL remains relatively low, with noticeably large pores in the mortar and weak bonding between gypsum crystals (Figure 9a). As shown in Figure 9b,c, the incorporation of KH-Al supplies an additional aluminum source for GSL, which reacts with sulfate released from by-product gypsum to form AFt. These hydration products collectively fill the mortar pores, and the interlocking and overlapping of different hydration products create a dense microstructure, thereby enhancing the mechanical properties of the mortar. However, when the KH-Al doping concentration reaches 1%, the sizes of AFt crystals become inconsistent, and large gypsum crystals appear locally (Figure 9d). This occurs because excessive KH-Al provides an abundant supply of active aluminum sources and a large reactive surface area. KH-Al reacts rapidly to generate a substantial amount of [Al(OH)_4_]^−^, accelerating the hydration rate of AFt and causing a sharp decrease in effective water content, which in turn inhibits the further hydration of CŜH_0.5_. The evolution of microstructure with curing time is intrinsically linked to the development of mechanical properties. As the hydration reaction progresses, the increased formation of hydration products (such as ettringite (AFt) and dihydrate gypsum (CŜH_2_)) fills the interstitial pores, leading to a refinement of the pore structure and a denser matrix. This microstructural densification, characterized by a decrease in average pore size and total porosity, reduces stress concentration sites and enhances the integrity of the mortar. Consequently, the compact microstructure observed in later curing stages directly correlates with the superior flexural and compressive strengths measured in mechanical tests.

**Figure 10:**
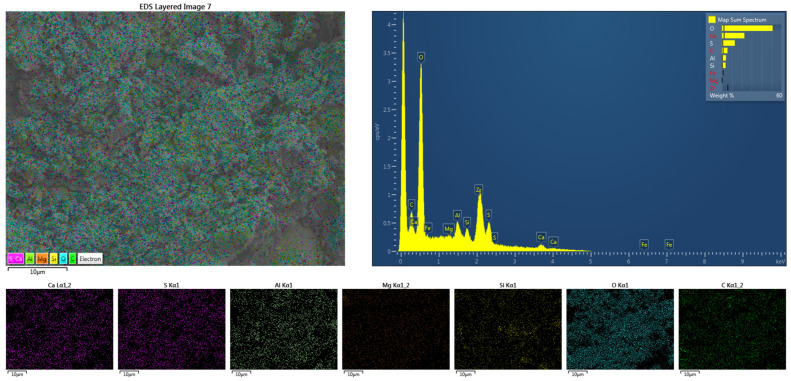
The EDS mapping of sample A4 after 28 days of curing.

Further EDS analysis was conducted on sample A4 to characterize the elemental distribution in GSL, as shown in Figure 10. The presence of O, Ca, S, Al, Si, along with minor amounts of Mg and C, was observed. Among these, O, Ca, and S originate from CŜH_2_; Al, Si, and Mg derive from SAC, FA, and KH-Al; while C comes from the alkyl chains in KH-Al. All elements are uniformly distributed without significant agglomeration. The GSL morphology appears as amorphous, densely packed aggregates, indicating that it primarily consists of CŜH_2_ gel material with substantial amounts of AFt gel filling the mortar pores. EDS analysis reveals high contents of sulfur and oxygen elements, along with rod-shaped or needle-like hydration products, suggesting that these formations result from reactions between KH-Al and sulfates under secondary activation [40]. As curing progresses, AFt gradually fills the pores within CŜH_2_, resulting in significantly enhanced mechanical properties of the GSL samples after 28 days of curing.

### 3.7. FT-IR Analysis

**Figure 11:**
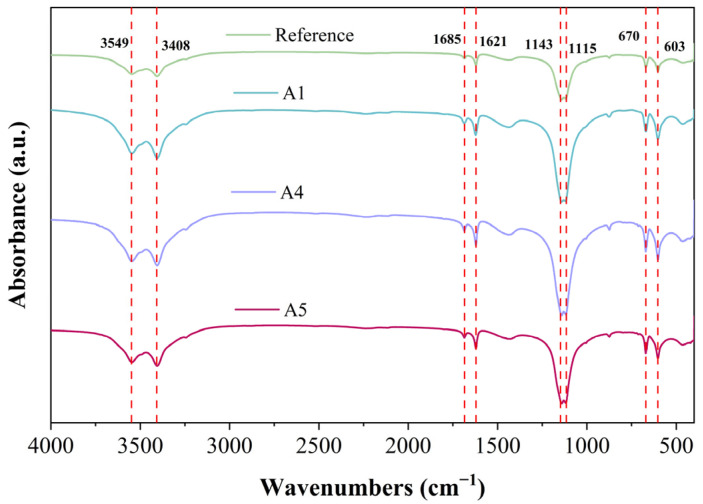
FT-IR spectra of mortar with different KH-Al content after 28 days of curing.

Figure 11 presents the FTIR spectra of various GSL samples after 28 days of curing. The peaks at 1143 cm^−1^ and 1115 cm^−1^ arise from the stretching vibrations of the S-O bond in the SO_4_^2−^ ions of gypsum dihydrate, while the peaks at 603 cm^−1^ and 670 cm^−1^ correspond to the asymmetric bending vibrations of the S-O bond in SO_4_^2−^ ions of gypsum dihydrate [41]. Compared to the control group, after the addition of KH-Al, the absorption peaks in the FTIR spectrum at the specified wavenumbers initially increase and then decrease. This trend indicates that excessive KH-Al inhibits the overall hydration process of gypsum, resulting in a reduced yield of hydration products. Conversely, an appropriate dosage of KH-Al promotes the hydration reaction, producing more hydration products. The absorption peaks at 3549 and 3408 cm^−1^ are attributed to the O–H stretching vibrations of H_2_O in dihydrate gypsum [42]. The peak at 1685 cm^−1^ corresponds to the bending vibration of H–O–H bonds in AFt [43]. The presence of these absorption peaks confirms substantial amounts of both physically adsorbed and bound water in the samples. As the KH-Al content increases, the peak widths and intensities across different bands initially increase and then decrease, indicating that the formation of hydration products alters the bound water content within the system. Regarding the aging behavior of the samples, no signs of carbonation were observed after 28 days of hydration. The absence of absorption peaks for carbonate ions (CO_3_^2−^) at 1420 cm^−1^ and 875 cm^−1^ suggests that the GSL effectively resists aging effects induced by time, humidity, and common atmospheric components such as CO_2_. These findings are consistent with the results observed in the previous XRD (Figure 6) and TG-DTG (Figure 7) analyses.

## 4. Conclusions

To improve the utilization rate of by-product gypsum, this study employs KH550-functionalized nano-activated alumina to modify GSL prepared from industrial by-product gypsum. The objective is to simultaneously enhance the mechanical strength and hydration kinetics of GSL. The main research findings are as follows:

1. The high specific surface area of KH-Al adsorbs free water, leading to a decrease in mortar fluidity. Meanwhile, it acts as nucleation sites that accelerate the hydration reaction, thereby shortening the initial and final setting times of GSL.
2. With the increase in KH-Al dosage, the mechanical properties of GSL improve significantly. At a dosage of 0.5%, the 24-h flexural strength and compressive strength of GSL increase by 42.83% and 45.89%, respectively, while the 28-day flexural strength and compressive strength increase by 70.86% and 78.12%, respectively.
3. KH-Al provides numerous nucleation sites for mortar hydration, increases the formation proportion of AFt and CŜH_2_, refines the crystal structure, and exhibits a significant hydration-promoting effect. Additionally, an appropriate dosage of KH-Al ensures a more uniform release of hydration heat throughout the reaction process, which benefits the development of later strength.
4. The SEM analysis results show that nanomaterials cause hydration products to interlock and overlap, filling pores and forming a dense structure. The FT-IR results are consistent with the XRD findings, confirming that KH-Al promotes the hydration reaction, whereas excessive KH-Al inhibits the overall hydration process.
5. The utilization of KH-Al modification enables the production of high-performance GSL, establishing a foundation for manufacturing high-value-added construction materials from industrial by-product gypsum. This approach paves the way for producing premium building materials from industrial gypsum by-products. For future industrial scalability, a cost–benefit analysis of employing surface-modified nanomaterials, as well as an assessment of the long-term durability of the modified GSL under real-service conditions, warrants further investigation. Nevertheless, the significant performance gains achieved at a relatively low dosage (0.5%) present a promising and potentially viable strategy for the advanced resource utilization of industrial solid waste.

## Figures and Tables

**Table 1:** Chemical composition of materials (wt%).

	SO_3_	CaO	SiO_2_	P_2_O_5_	Al_2_O_3_	K_2_O	MgO	Fe_2_O_3_	Na_2_O	TiO_2_	LOI
β-PG	49.63	35.97	6.12	0.29	0.41	0.16	0.11	0.08	0.09	0.06	7.18
β-FG	52.79	39.31	3.42	0.04	1.37	0.14	0.67	0.15	0.11	0.06	1.94

**Table 2:** Mix proportions of GSL.

Sample No.	Binder (%)			Aggregate (%)	Mineral Admixture (%)	Nanomaterials		Additives (%)			
	β-PG	β-FGD	SAC	MS	FA	Type	wt%	PCE	PR	HPMC	DA
Reference	20	40	10	15	15	-	-	0.3	0.04	0.04	0.08
A1	20	40	10	15	15	KH-Al	0.05	0.3	0.04	0.04	0.08
A2	20	40	10	15	15	KH-Al	0.1	0.3	0.04	0.04	0.08
A3	20	40	10	15	15	KH-Al	0.25	0.3	0.04	0.04	0.08
A4	20	40	10	15	15	KH-Al	0.5	0.3	0.04	0.04	0.08
A5	20	40	10	15	15	KH-Al	1	0.3	0.04	0.04	0.08

**Table 3:** Proportions of hydration products in different GSL samples at 24 h and 28 days.

Sample No.	24 h				28-Day			
	AFt Mass Loss/%	CŜH_2_ Mass Loss/%	AFt/%	CŜH_2_/%	AFt Mass Loss/%	CŜH_2_ Mass Loss/%	AFt/%	CŜH_2_/%
Reference	0.68	10.49	2.37	50.12	0.63	10.41	2.19	49.74
A1	1.42	10.65	4.95	50.89	1.48	11.07	5.16	52.90
A2	1.86	11.06	6.48	52.85	1.63	10.91	5.68	52.13
A3	2.13	11.21	7.42	53.56	1.86	10.89	6.48	52.04
A4	2.31	11.56	8.05	55.24	2.08	10.86	7.25	51.89
A5	1.97	10.96	6.86	52.37	1.69	10.58	5.89	50.56

## Data Availability

The original contributions presented in this study are included in the article. Further inquiries can be directed to the corresponding author.
